# Role of magnesium-doped calcium sulfate and β-tricalcium phosphate composite ceramics in macrophage polarization and osteo-induction

**DOI:** 10.1007/s10266-022-00708-6

**Published:** 2022-06-02

**Authors:** Jing Zhou, Su Sun, Yan He, Tingting Yan, Jianfeng Sun, Jie Pan, Shuyu Zhu, Liqiong Chen, Pengfei Zhu, Biao Xu, Yan Liu

**Affiliations:** 1 The Affiliated Stomatology Hospital of Kunming Medical University, No. 1088 Haiyuan Middle Road, Kunming, 650500 Yunnan China; 2 Department of Stomatology, Kunming Yanan Hospital, Kunming, 650051 China; 3 Key Laboratory of Tumor Immunological Prevention and Treatment of Yunnan Province, Kunming, 650051 China; 4 Department of Stomatology, Kunming Municipal Hospital of Traditional Chinese Medicine, Kunming, China; 5 Laboratory for Regenerative Medicine, Tianyou Hospital, Wuhan University of Science and Technology, Wuhan, 430064 China; 6 Faculty of Material Science and Engineering, Kunming University of Science and Technology, Kunming, China; 7 Department of Orthodontics, Ningbo Stomatological Hospital, Ningbo City, Zhejiang Province China; 8 Department of Stomatology, The First People’s Hospital of Yunnan Province, Kunming, China

**Keywords:** Osteoinduction, Macrophage polarization, CaSO_4_/β-TCP, Flow cytometry, Western blot

## Abstract

In the current study, we explored the role of Mg^2+^-doped CaSO_4_/β-TCP composite biopolymer in regulating macrophage polarization and its relation with enhanced osteogenic differentiation of periodontal ligament stem cells. Furthermore, mechanism underling the regulation of macrophage polarization by CaSO_4_/β-TCP was evaluated. Mg^2+^-doped CaSO_4_/β-TCP composite was characterized by scanning electron microscopy (SEM) and X-ray diffraction (XRD). Macrophage polarization was characterized using flow cytometry analysis. Macrophage morphometric analysis was conducted by FITC phalloidin staining. Western blot and qRT-PCR assays were used to assess gene expression levels and miRNAs, respectively. SEM morphology of CaSO_4_/β-TCP ceramic revealed a particle size of 10–50 μm, and XRD spectrum showed that characteristic peak of samples was consistent with that of CaSO_4_ and β-TCP. Results from flow cytometry evidenced significant upregulation of M2 macrophage markers after adding ceramic biopolymer, indicating the induction of inactivated M0 macrophage polarization to M2 macrophage. Macrophage morphometric analysis revealed development of lamellar pseudopodia on day 7 in CaSO_4_/β-TCP group. Furthermore, flow cytometry revealed high positivity rate of 90.34% (CD44) and 89.36% (CD146). qRT-PCR results showed that the level of miR-21-5p was significantly decreased in M2 macrophages. Moreover, western blot analysis revealed upregulated expression levels of RUNX2, osterix (Osx), and osteopontin (OPN), and ELISA exhibited increase in cytokine levels (IL-1β, IL-10, TGF-β1, and BMP-2) in the presence of macrophages, indicating the osteogenic differentiation ability of periodontal ligament stem cells. The study evidenced the regulation of macrophage polarization by Mg^2+^-doped CaSO_4_/β-TCP composite ceramic and its mediation through lncRNA PVT1/miR-21-5p/smad2 molecular axis.

**Supplementary Information:**

The online version contains supplementary material available at 10.1007/s10266-022-00708-6.

## Introduction

Periodontitis represents the most common inflammatory oral disease characterized by chronic inflammation of the periodontium and the surrounding soft tissues that results in the degradation of periodontal tissue, eventually leading to the loss of tooth and bone if left untreated [1]. Recent studies substantiated that the prevalence and severity of periodontal disease (PD) is very high among adults in mainland China [2]. Furthermore, conventional therapies (such as supragingival scaling, subgingival scaling, and root planning) for PD aim to eliminate the infectious sources, reduce inflammation, and arrest the disease progression [3]. However, these modalities cannot achieve ideal repair and regeneration of lost periodontal tissue specifically in patients with advanced PD [3]. Therefore, several periodontal regeneration therapies, such as guided tissue regeneration, growth factor delivery, enamel matrix derivative, bone grafts, and matrix-based scaffolds containing combination of cells and growth factors, have been developed to target the periodontal regeneration in patients with advanced PD. However, these intervention methods involve complex procedures and cannot achieve complete regeneration of periodontal tissue, and the clinical outcomes of those approaches are variable and unpredictable [3, 4].

Recently, several studies substantiated the osteo-induction potential of several tissue engineering biomaterials (e.g., collagen, hydroxyapatite, biphasic calcium phosphate, tricalcium phosphate, calcium pyrophosphate, alumina ceramics, and porous titanium) without exogenous cells and growth factors [5]. Within this broad class of osteoinductive ceramics, composites of calcium sulfate and beta-tricalcium phosphate (CaSO_4_/β-TCP) are recently reported to have enhanced biocompatibility and osteogenic and biodegradation properties [6]. Furthermore, existing evidence suggests that macrophages (M1 and M2 phenotypes) are the key modulators that evoke the inflammatory response after biomaterial implantation and induce osteogenic differentiation by modulating the cellular pathways such as integrin–mitogen-activated protein kinase (MAPK) pathway and the bone morphogenic pathway (BMP) *Caenorhabditis elegans* Sma genes and the *Drosophila* Mad (SMAD2) pathway [7–9]. However, the exact mechanism of the pathways that influence tissue regeneration and its regulation is yet to be elucidated. Furthermore, recent studies evidenced that Mg ions (Mg^2+^) could promote osteogenic differentiation of periodontal ligament stem cells, regulate the immune function of macrophages, stimulate adhesion of macrophages, and promote M2 polarization of macrophages [10].

Therefore, the current study aimed to explore the role of Mg^2+^-doped CaSO_4_/β-TCP composite biopolymer in regulating macrophage polarization and its relation with enhanced osteogenic differentiation of periodontal ligament stem cells. The mechanism underling the regulation of macrophage polarization by CaSO_4_/β-TCP was also evaluated.

## Materials and methods

### Fabrication and characterization of Mg^2+^-doped CaSO_4_/β-TCP ceramics

Mg^2+^**-**doped β-TCP was prepared using the precipitation method. Briefly, an aqueous solution of predetermined proportions of CaCO_3_ and MgCO_3_ was prepared and phosphoric acid was added rapidly to the above solution and stirred continuously for 12 h to make the reaction complete. The nominal composition in terms of (Ca + Mg)/P ratio was maintained at 1.50. Later, the precursor of Ca_3_(PO_4_)_2_ was filtered and dried in oven at 80 ℃ for 12 h. Following this, the precursor was subjected to air sintering in muffle furnace for 6 h (heating rate: 5 ℃/min; heated to 1100 ℃) and air cooled. Furthermore, sintered β-TCP was grounded using planetary ball mill, sieved (200 mesh), and sealed in a bag. The biologic scaffold of Mg^2+^-doped CaSO_4_/β-TCP was prepared by mixing β-TCP and CaSO_4_ (ratio: 1:7) in deionized water (2.5 g/L), and the slurry was filled into a 50-well plate, sealed, and hydrated in an oven at 40 ℃ for 24 h and then dried for 24 h. Furthermore, microtopography and phase composition of the prepared ceramics were analyzed using scanning electron microscopy (SEM) and X-ray diffraction (XRD), respectively**.**

### Culturing and seeding of macrophages

Human THP-1 cells were maintained in RPMI-1640 (88%) supplemented with fetal bovine serum (FBS; 10%), double antibody, as well as glutamine (1%) and incubated with 5% CO_2_ at 37 °C. Cells were inoculated into 6-well plate at a density of 7 × 10^4^ cells/well. Later, phorbol myristate acetate (100 ng/mL) was added to induce macrophages and subsequent experiments were carried out. CaSO_4_/β-TCP ceramic copolymer was co-cultured with stem cells, and the differentiation was observed using transmission electron microscope (TEM) after primary culture.

### Isolation, culture and identification of periodontal ligament stem cells

Orthodontic-extracted disease-free premolar teeth of patients aged 10–15 years were collected and washed three to five times using Dulbecco’s phosphate-buffered saline (DPBS). The middle-third of the roots were scraped gently from the periodontal ligament tissue. The tissue obtained were minced into smaller pieces of approximately 1 mm^3^. Enzymatic digestion was employed using freshly prepared 3 mg/ml collagenase type I solution and incubated at 37 °C in 5% CO_2_ for 45 min. The enzymatic action of collagenase was neutralized by adding equal amount of Dulbecco’s modified eagle’s medium (DMEM) and the mixture was then passed through a 70 µm strainer to prepare single-cell suspension which was then transferred to DMEM containing 10% fetal bovine serum, 1% penicillin–streptomycin and incubated in humidified atmosphere of 37 °C and 5% CO_2_ for three days. The medium was changed every three days. Floating cells were removed after 72 h and the adherent cells were further expanded. Experiments were performed using either passage(P) 3 cells. The purified periodontal ligament stem cells were amplified, treated with an appropriate amount of protease and cleaned with PBS. The cell cycle was detected by flow cytometry. All the procedures were conducted at the Key Laboratory of Tumor Immunological prevention and Treatment of Yunnan Province. Further, all the experimental procedures were approved by the medical ethics committee of Kunming Yan'an Hospital (Approval number: 2021-08-03-001).

### In vitro osteogenic differentiation potential of human periodontal ligament stem cells

Successfully cultured third-generation periodontal ligament stem cells were co-cultured with osteogenic inducer, macrophage-conditioned medium (ratio of macrophage-conditioned medium to osteogenic inducer: 1:2), and CaSO_4_/β-TCP composite ceramic polymer + macrophage-conditioned medium (ratio of polymer + macrophage-conditioned medium to osteogenic inducer: 1:2) for 14 days. Alizarin red staining was used to identify the osteogenesis and alkaline phosphatase (ALP) assay was used to monitor the osteogenic differentiation of periodontal ligament stem cells. ALP assay was conducted using CDP-star assay kit and the assay was carried out according to the manufacturer’s instructions.

### Enzyme-linked immunosorbent assay

To investigate the effects of Mg^2+^-doped CaSO_4_/β-TCP on the secretion of cytokines and chemokines in macrophage, Mg^2+^-doped CaSO_4_/β-TCP scaffolds were inoculated with THP-1 cells, and after 3 days, the supernatant was collected. The levels of the cytokines (Interleukin-1β (IL-1β) and Interleukin-10 (IL-10) and chemokines (Transforming growth factor-β1 (TGF-β1) and bone morphogenetic protein (BMP-2) in the supernatant were measured using commercially available enzyme-linked immunosorbent assay (ELISA) kits (BMP-2 [Elab science, E-EL-H0011c]; IL-1β [ml058059]; IL-10 [ml064299]; TGF-β1 [Elabscience, E-EL-0162c]; AKP [Nanjing Jiancheng Institute of Biological Engineering, A059-2]) according to the manufacturer’s instructions. The absorbance was detected at 450 nm using a microplate reader (SPECTCA MAX190).

### Flow cytometry

Macrophage polarization was characterized using flow cytometry analysis. Briefly after 3 days of culture, cells were harvested with 0.25% trypsin, washed with phosphate-buffered saline (PBS), and washed and resuspended in Stain Buffer with FBS to avoid nonspecific antibody binding. Following the manufacturer’s instructions, cells were incubated with monoclonal antibodies, including cluster of differentiation 68 (CD68), cluster of differentiation 206 (CD206) monoclonal antibodies, inducible nitric oxide synthase (iNOS) monoclonal antibodies, and arginase, at 4 °C for 30 min. Three replicas were included in each group. Signals were detected with a flow cytometer, and the data obtained were analyzed.

### Western blot assay

Cells were washed twice using PBS and lysed using radioimmunoprecipitation assay buffer (RIPA) buffer containing 30 μL protease inhibitor and subjected to centrifugation at 4 ℃ and 12,000 rpm for 10 min, and the supernatant was collected. After this, the total protein concentration was quantified using bicinchoninic acid (BCA) assays using BCA Protein Assay Kit. Later, the lysate was denatured, separated by standard sodium dodecyl sulfate–polyacrylamide gel electrophoresis (SDS-PAGE; (4% SDS-PAGE, 60 V for 25 min and 90 V for 3 h) and then transferred onto a polyvinylidene difluoride (PVDF) blotting membrane (1 h at 200 mA) and the membrane was blocked with 5% bovine serum albumin and sealed at room temperature (20–30℃) for 1 h. The membranes were incubated with primary antibodies against rabbit anti-Smad2 antibody (BS0718R, 100 uL, Bioss) overnight at 4℃. Glyceraldehyde-3-phosphate dehydrogenase (GADPH) was used as a loading control. After washing with tris-buffered saline (TBS) buffer, the membranes were incubated with horse radish peroxidase–conjugated secondary antibodies for 1 h at room temperature. The protein bands were visualized using an electrochemiluminescence (ECL) solution, and the relative intensity was quantified.

### Semiquantitative real-time PCR test

Quantitative reverse transcription PCR (qRT-PCR) assay was used to assess the effects of ceramic properties on the gene expression levels of the abovementioned cytokines and chemokines. Total RNA was isolated using Trizol Reagent kit (Lifetech 15596026) and subsequently reverse transcribed to complementary DNA (cDNA) using the FastKing cDNA first strand synthesis kit (KR116). A real-time polymerase chain reaction (RT-PCR) reaction was carried out using LightCycle 96. Furthermore, the relative expression of each targeted gene, including CD68, CD206, iNOS, arginase, and smad2 (primers are listed in Table1), was calculated and normalized using $2^{{ - \Delta \Delta C_{{\text{t}}} }}$ method. GAPDH served as a housekeeping gene and used for the relative quantification.

**Table 1:** Primer pairs used in semiquantitative real-time PCR

Gene	Primer sequence		Length (bp)
GAPDH	Forward	AAAGGGTCATCATCTCTG	80
	Reverse	GCTGTTGTCATACTTCTC	
iNOS	Forward	ATATTACGGCTCCTTCAA	83
	Reverse	CTGTTGTTTCTATCTCCTTT	
CD68	Forward	AGACCATTGGAGACTACA	82
	Reverse	TACATGACTCGAATCTGAAT	
CD206	Forward	CTACTATGTCTTGGAATGATAT	120
	Reverse	TAACTGGTGGATTGTCTT	
Arginase	Forward	AAGACACCAGAAGAAGTA	96
	Reverse	AATAGGCTTGTGATTACC	
smad2	Forward	ATTGAGCCACAGAGTAAT	135
	Reverse	AAGAGTAGTAGGAGATAGTTC	

*bp* base pair, *CD68* cluster of differentiation 68, *CD206* cluster of differentiation 206, *iNOS* inducible nitric oxide synthase, *GADPH* glyceraldehyde-3-phosphate dehydrogenase

### Statistical analysis

The experiments were performed with at least 3 replicates, and all the results were represented as mean values ± standard deviation (SD). An independent sample *t* test was used when comparisons were performed between the 2 groups, and 2-way analysis of variance (ANOVA) was performed for multiple comparison. All the analysis were performed using GraphPad Prism (GraphPad Software Inc., La Jolla, CA, USA). *P* values < 0.05 were considered statistically significant.

## Results

### Characterization of Mg^2+^-doped CaSO_4_/β-TCP ceramics

**Fig. 1:**
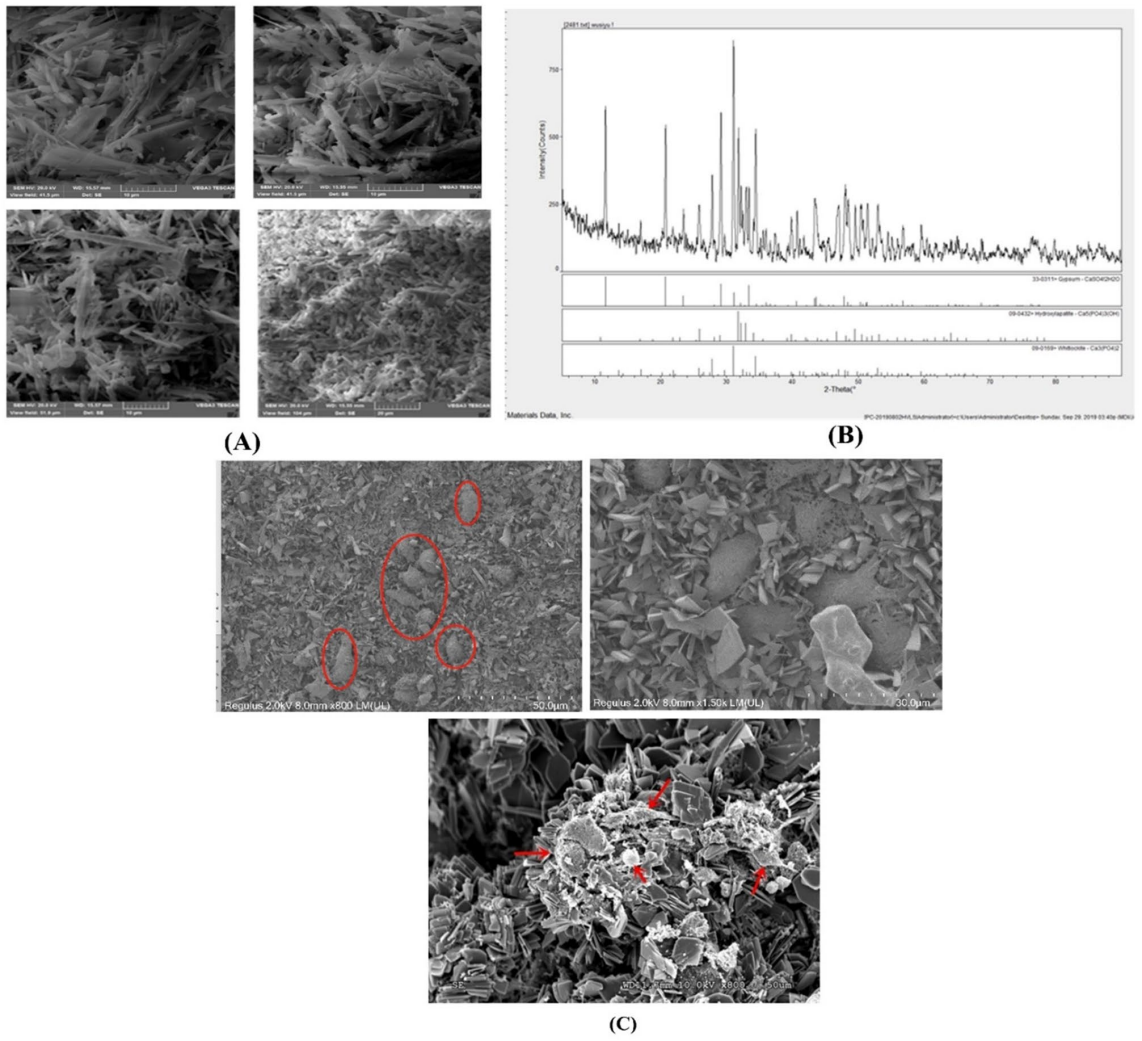
Characterization of CaSO_4_/β-TCP ceramic composite. **A** SEM morphology of CaSO_4_/β-TCP ceramic composite. **B** XRD graphs. **C** TEM images of macrophages grown on CaSO_4_/β-TCP ceramic composite

SEM morphology of CaSO_4_/β-TCP ceramic composite revealed a particle size of 10–50 μm. Furthermore, the ratio of length to diameter of the whisker decreased and the flake structure on the surface of the powder disappeared and became smooth (Fig. 1A). The XRD spectrum shows that the peak of the sample is consistent with the characteristic peak of CaSO_4_ and β-TCP. Moreover, the characteristic peak of β-TCP when 2*θ* is 77°, 31.02°, and 34.37° with corresponding crystal faces are (214), (0210), and (220), respectively. The diffraction peaks near 2*θ* of 31.77°, 32.19°, and 32.9° correspond to the characteristic peaks of hydroxyapatite. By comparing the XRD spectrum of CaSO_4_, the diffraction peaks near 11.58° and 20.72° are completely consistent with the characteristic peaks of CaSO_4_·2H_2_O (Fig. 1B).

### Identification of macrophages

TEM images after primary culture revealed that the macrophages were irregular in shape and the nucleus was dark blue, mostly distorted to 1 side representing a typical morphological characteristic of macrophages (Fig. 1C).

### In vitro analysis of macrophage morphology and polarization and induced by CaSO_4_/β-TCP

**Fig. 2:**
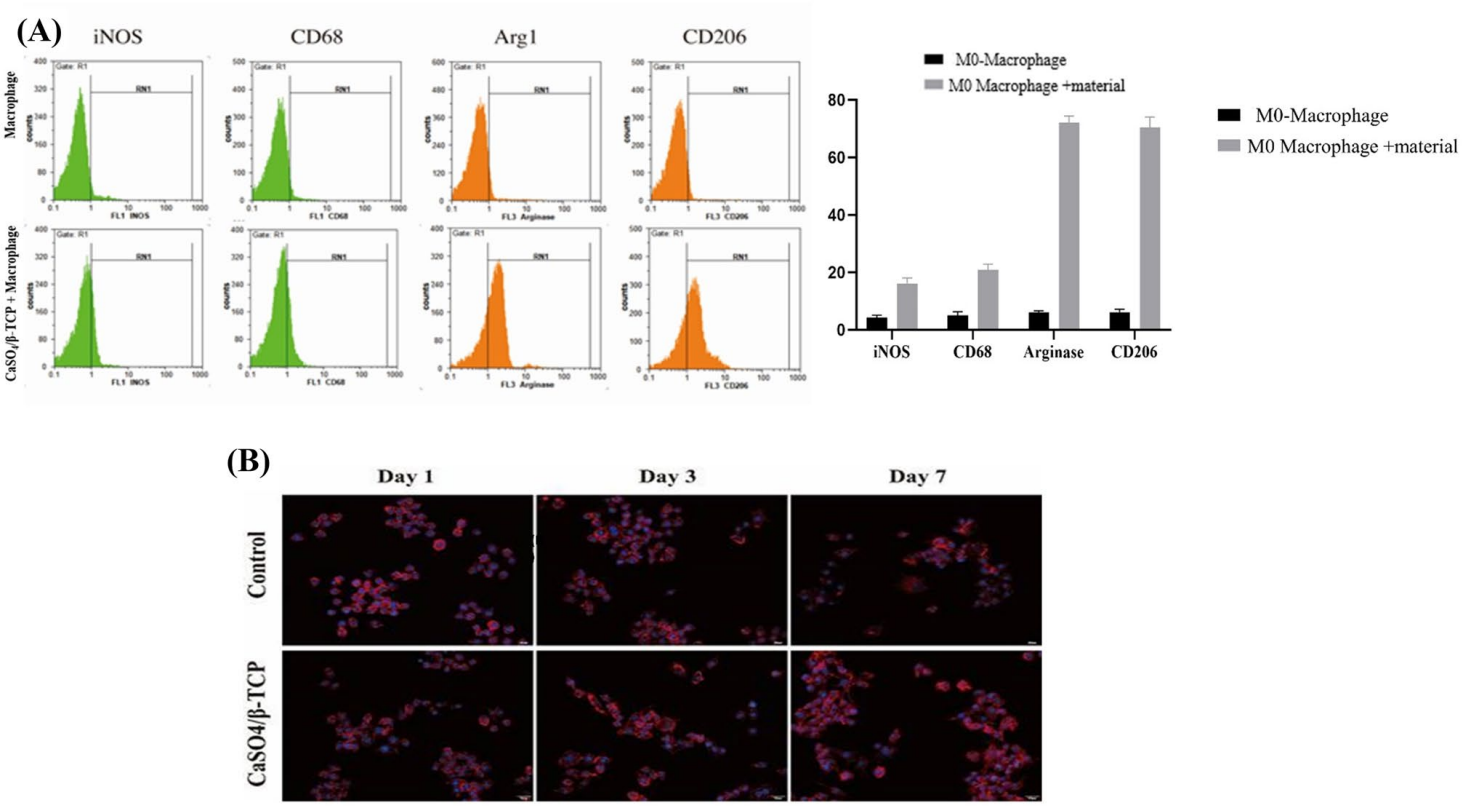
**A** Evaluation of macrophage polarization regulated by CaSO_4_/β-TCP ceramic composites by flow cytometric analysis. **B** Macrophage morphology of control and (CaSO_4_)/β-TCP co-cultured cells by FITC phalloidin staining. *CD68* cluster of differentiation 68, *CD206* cluster of differentiation 206, *iNOS* inducible nitric oxide synthase, *GADPH* glyceraldehyde-3-phosphate dehydrogenase

After co-culturing CaSO_4_/β-TCP with non-activated M0 macrophages, the results of flow cytometry demonstrated that the expression of M2 macrophage markers (arginase and CD206) was significantly upregulated after adding CaSO_4_/β-TCP ceramic biopolymer, indicating that CaSO_4_/β-TCP composite ceramic materials induce inactivated M0 macrophage polarization to M2 macrophage (Fig. 2a). Furthermore, the morphometric analysis by Fluorescein isothiocyanate (FITC) phalloidin staining for both control cells and CaSO_4_/β-TCP co-cultured cells shows that the cells in the 2 groups were round on the first day; on the third day, the cell morphology began to show significant differences, where the control group predominantly showed M1 phenotype, whereas a typical M2 spindle phenotype was observed in the CaSO_4_/β-TCP group; on the seventh day, the pseudopodia of the control group were well developed in the shape of a "star," and in the CaSO_4_/β-TCP group, the lamellar pseudopodia extended to the cells and the spindle phenotype was seen more significantly (Fig. 2b).

### Identification of periodontal ligament stem cells

The primary periodontal ligament stem cells were isolated and cultured, and the phenotype expression of periodontal ligament stem cells was identified using flow cytometry. The cell surface markers, CD44 and CD146, showed a high positivity rate of 90.34 and 89.36%, respectively, whereas the lack of expression was observed for hematopoietic markers CD34 and CD45 (Online Resource 1).

### Validation of preselected gene

After co-culturing CaSO_4_/β-TCP composite ceramic materials with inactivated M0 macrophages, the cells were collected and the protein was extracted. The expression of smad2 protein was detected by western blotting (WB). The results showed that the expression levels of smad2 protein were upregulated after adding the CaSO_4_/β-TCP ceramics, suggesting that the biopolymer promoted the polarization of macrophages (Online Resource 2). Furthermore, the mRNA levels of M1 macrophage activation markers (iNOS and CD68) and M2 macrophage activation markers (arginase and CD206) were detected using the in vitro RT-qPCR assay. The results showed that upregulation of the macrophage activation markers increased with significant upregulation of M2 macrophage activation markers (arginase and CD206; Online Resource 3).

**Fig. 3:**
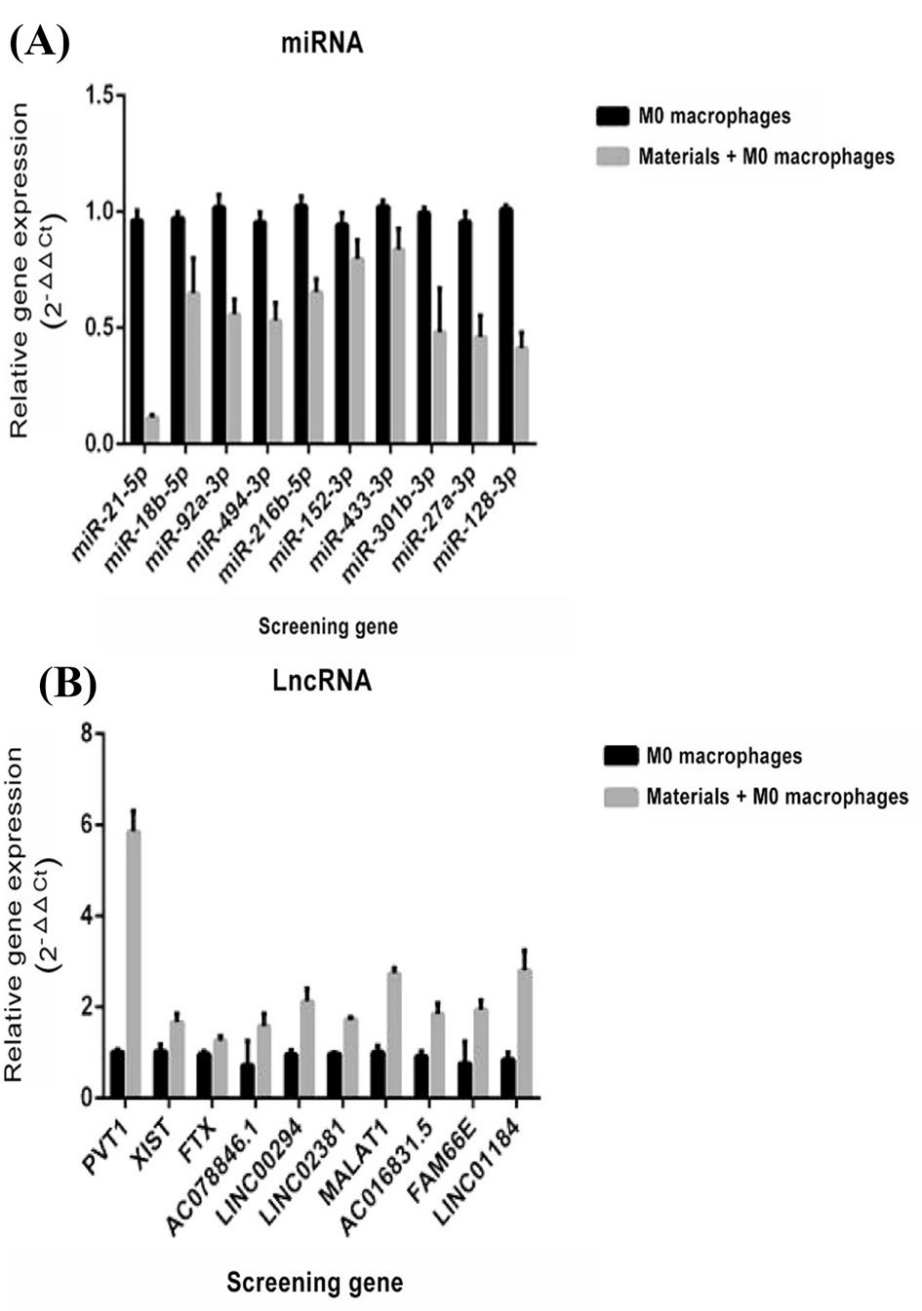
Relative gene expression of **A** miRNA and **B** lncRNA candidates using qRT-PCR

On the basis of the results of the proteome profiler cytokine array and other earlier reported evidences, 10 miRNAs and lncRNAs secreted by macrophages were identified for further testing. Semiquantitative in vitro RT-qPCR assay showed down-regulation of 10 miRNA candidate genes, namely, miR-21-5p, miR-18b-5p, miR-92a-3p, miR-494-3p, miR-216b-5p, miR-152-3p, miR-433-3p, miR-301b-3p, miR-27a-3p, and miR-128-3p, of which the expression of niR-21-5p gene was significantly reduced when compared with the expression levels of housekeeping GAPDH (Fig. 3a). Similarly, among the previously identified candidate lncRNAs for macrophage polarization, PVT1, XIST, FTX, AC078846.1, lnc00294, lnc02381, MALAT1, AC016831.5, FAM66E, and Lnc01184 lncRNAs showed upregulation after the addition of CaSO_4_/β-TCP ceramic polymer in the co-cultured media; the expression of PVT1 gene was significantly upregulated (Fig. 3b). Therefore, miR-21-5p and PVT1 were confirmed as a preselected candidate mRNA aiding in macrophage polarization.

### In vitro effects on cytokine expression in macrophages

**Fig. 4:**
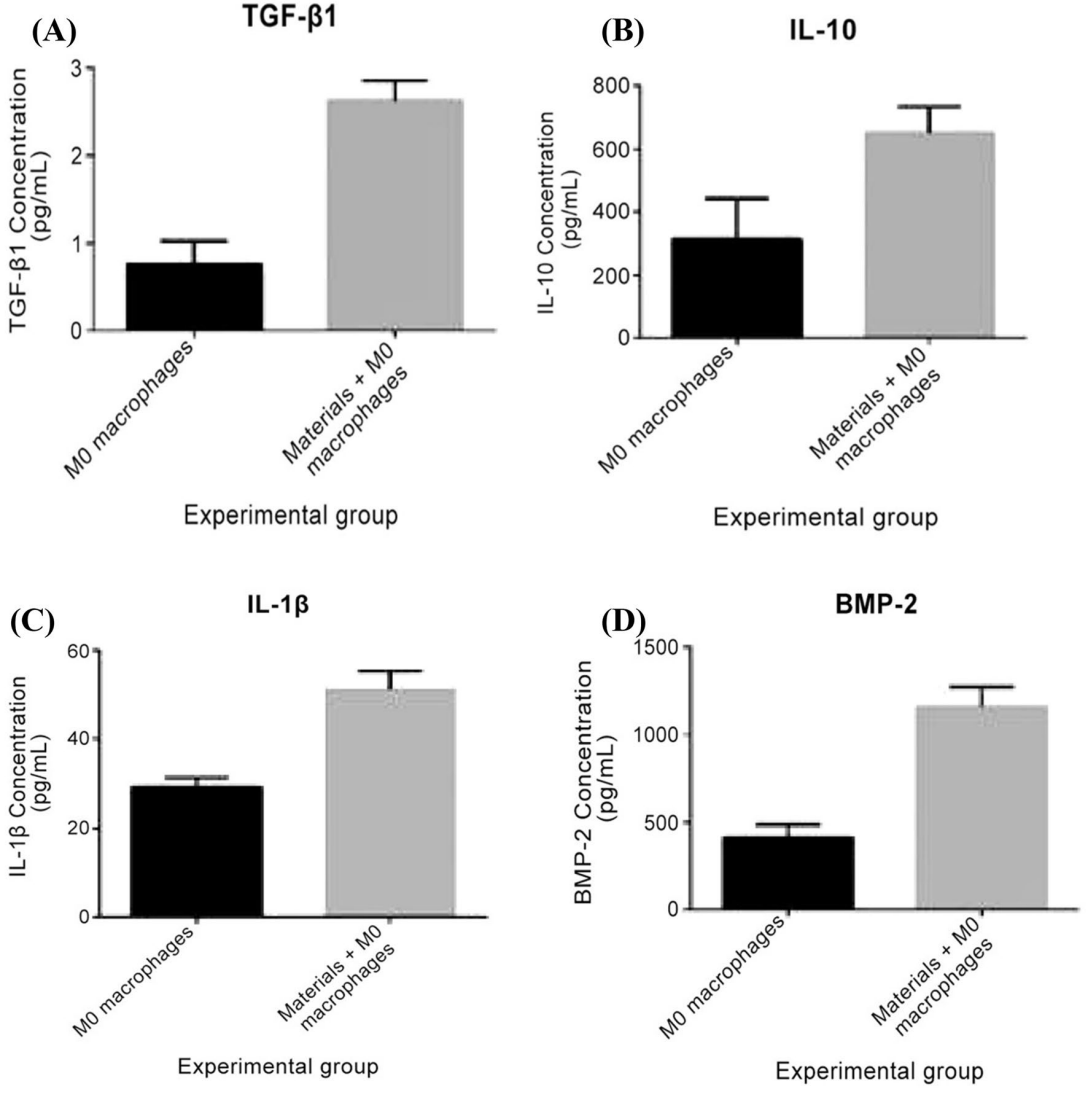
Changes in cytokine concentration levels detected by ELISA: **A** transforming growth factor β1, **B** interleukin 10, **C** interleukin 1β, and **D** bone morphogenic protein 2

To investigate the effects of phase composition on macrophage secretion, a commercial antibody ELISA assay was used to screen for inflammation-associated cytokines (IL-1β and IL-10) and chemokines (TGF-β1 and BMP-2) in the supernatants. As shown in Fig. 4, IL-1β, IL-10, TGF-β1, and BMP-2 cytokine concentration levels were significantly enhanced in the co-cultured medium after the addition of the CaSO_4_/β-TCP ceramic polymer when compared with the culture containing only the macrophages, which suggests that the biopolymer could promote the polarization of M0 macrophages.

### Osteogenic differentiation of periodontal ligament stem cells

**Fig. 5:**
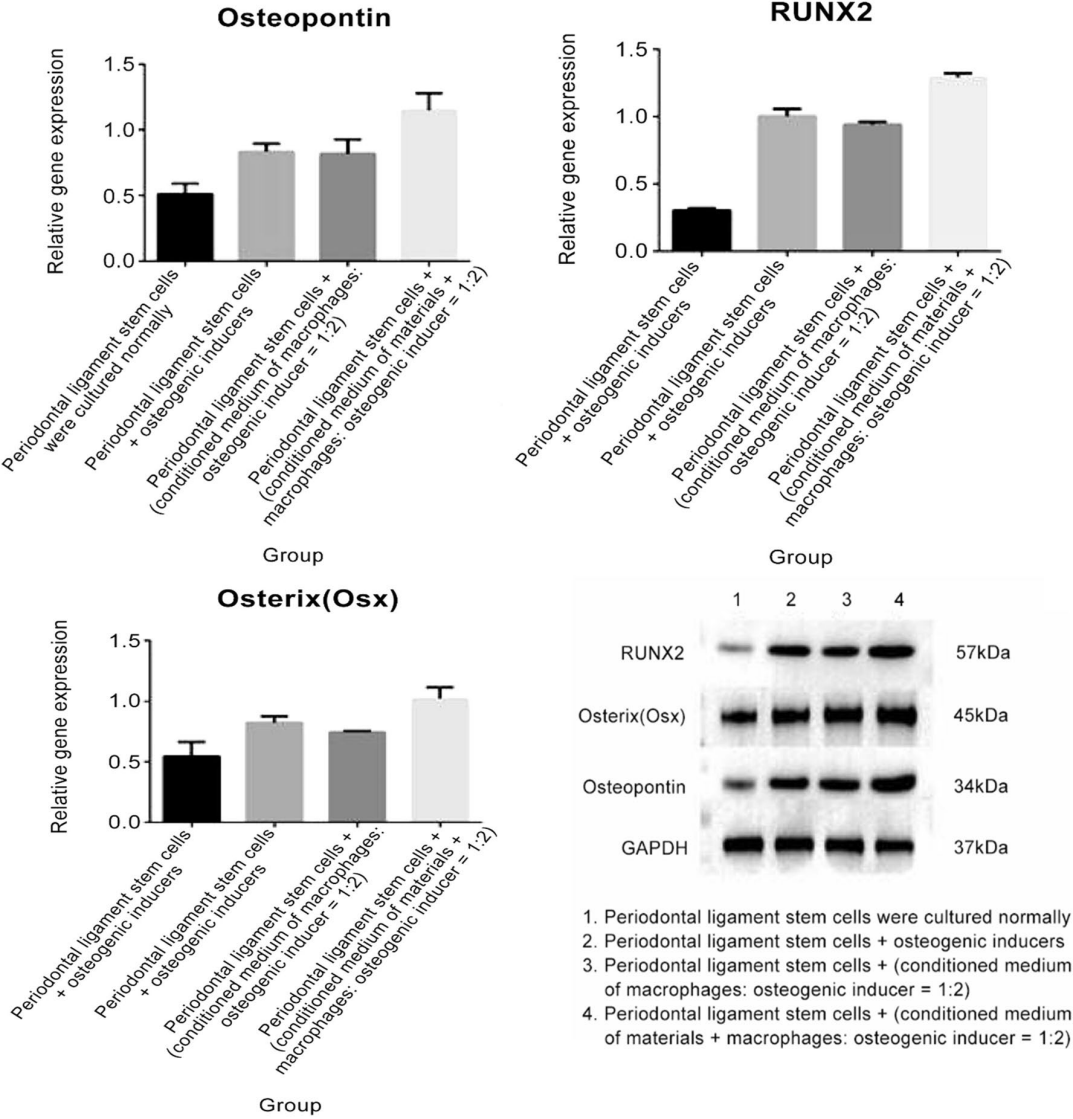
Western blot analysis and quantitative gene expression of the fold change of osteopontin, osterix, and runX2 proteins as compared with that of housekeeping GAPDH protein

**Fig. 6:**
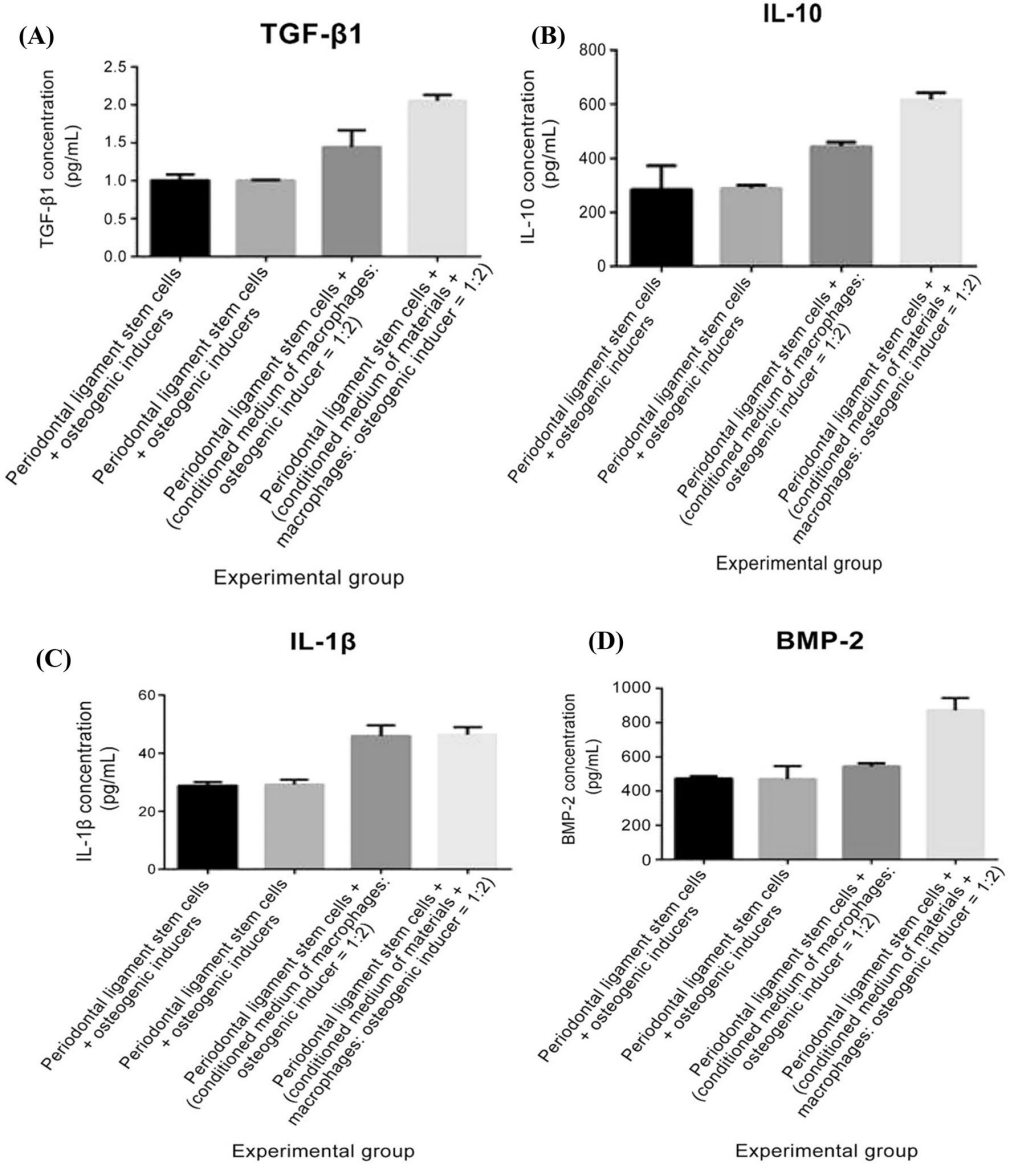
Changes in cytokine levels as detected by ELISA for periodontal stem cell in conditioned media ELISA: **A** transforming growth factor β1, **B** interleukin 10, **C** interleukin 1β, and **D** bone morphogenic protein 2

To investigate the effects of macrophage secretory factors on osteogenesis, the conditioned media from the macrophage/calcium phosphate supernatants were used to culture stem cells. In the presence of macrophages, the osteogenic differentiation ability of periodontal ligament stem cells increased significantly. The ALP activity was increased in the periodontal ligament stem cells + (conditioned medium of material + macrophage + osteoinducer [1:2]) experimental group but not in the other experimental groups (Online Resource 4). Furthermore, positive upregulated expression levels of RUNX2, osterix (Osx), and osteopontin (OPN) when compared with the GAPDH controls were detected using western blot (Fig. 5). Furthermore, the supernatant from both the co-cultured cells (with and without biopolymer) was collected, and the protein was extracted; IL-1β, IL-10, TGF-β1, and BMP-2 cytokine concentration levels were detected using ELISA (Fig. 6). The results obtained evidenced the increase in cytokine levels in the presence of macrophages. Furthermore, the results show that the osteogenic differentiation ability of periodontal ligament stem cells is significantly upregulated in the presence of macrophages in the conditioned media. These results suggest that the material can promote the osteogenic differentiation of periodontal ligament stem cells by influencing the polarization of macrophages and promoting the secretion of cytokines beneficial to osteogenic differentiation.

## Discussion

Earlier, several studies substantiated the osteoinduction capability of biomaterials [11]. However, the exact nature of induction, subsequent differentiation, and its regulation remain unknown [12]. The results obtained from the current study substantiated that Mg^2+^-doped CaSO_4_/β-TCP composite ceramic material might promote the osteogenic differentiation of periodontal ligament stem cells by activating M2 macrophages and secreting a large number of cytokines.

Recent reports have indicated the indispensable role of macrophages in regulating bone formation in non-osseous sites in tissue damage [9, 13]. Furthermore, earlier studies evidenced that the change in polarization balance of M1/M2 macrophages can control the fate of organs during inflammation or injury [14]. When infection or inflammation is severe enough to affect organs, macrophages first show M1 phenotype and release TNF-α, IL-12, and IL-23 antagonized stimulation [7]. However, if the M1 phase continues, it will lead to subsequent tissue damage. Therefore, M2 macrophages secrete increased levels of IL-10 and TGF-β to inhibit the inflammatory reaction; promote tissue repair, remodeling, and angiogenesis; and maintain homeostasis [15]. The results obtained from the present study are in agreement with those from previous literature. Our results showed that the proportion of M2 type macrophages was significantly increased in the co-culture of CaSO_4_/β-TCP composite ceramic material and with significant upregulation of IL-1β, IL-10, TGF-β1, and BMP-2 cytokine expression levels.

A recent study by He et al. [16] and Jin et al. [17] also demonstrated that in the presence of IL-4, macrophages are recruited to transplanted gels and likely to polarize to the immunoregulatory M2 state. Therefore, when these cells are stored in an indirect or direct co-culture system, they positively affect the osteogenic differentiation of bone mesenchymal stem cells. The incorporation of magnesium ions (Mg^2+^) in the biopolymers reduces macrophage inflammatory factors such as TNF-α, whereas the expression of IL-6 and IL-10 induces macrophages to hypothesize to express BMP-2 and TGF-β1 cytokines [9]. BMP-2 secreted by M2 macrophages has shown to be a critical factor in promoting the osteogenic differentiation of periodontal ligament stem cells, and studies have confirmed that BMP-2 has a significantly high osteogenic effect [18]. This observation is in accordance with our results where BMP-2 cytokine concentration levels were significantly enhanced in the co-cultured medium after adding the CaSO_4_/β-TCP ceramic polymer compared with the culture containing only the macrophages, which suggests that the biopolymer could promote the polarization of inactivated M0 macrophages.

Numerous preclinical studies have shown that the expression of smad2 protein is correlated with M2 macrophages [9, 19]. In myofibroblasts, M2 macrophages are activated, leading to increased secretion of TGF-β, which leads to the activation of the TGF-β/smad2 signaling pathway; in mouse models of pulmonary fibrosis, the increase in M2 macrophages was accompanied by the increase in TGF-β1 and p-smad2/3 proteins [20, 21]. Furthermore, in vitro studies have shown that in co-cultures of M2 macrophages and MLE-12 cells, the TGF-β1 and p-smad2/3 upregulation smad1/5-mediated signaling was correlated with BMP stimulation [22]. The results obtained in the study are in accordance with the literature; the results showed that the expression levels of smad2 protein were upregulated after adding the CaSO_4_/β-TCP ceramics, suggesting that the biopolymer promoted the polarization of macrophages.

Another noteworthy point substantiated in the present study is the involvement of miRNA in regulating the macrophage polarization in periodontal cells. Earlier studies demonstrated that the polarization of M1 macrophages requires the regulation of miRNA-125 [23], miRNA-146, miRNA-155, miRNA-let-7a/f, and miRNA-378 [24]; consequently, M2 polarization requires the activation of miRNA-let-7c/e, miRNA-9, miRNA-146, miRNA-147, miRNA-187, and miRNA-223 [25]. Zhao et al. has shown that miR-21-5p can promote inflammatory response and subsequently activate M1 macrophages [26]. The results of semiquantitative RT-PCR from the present study revealed that the level of miR-21-5p was significantly decreased in M2 macrophages in accordance with earlier evidences. Similarly, lncRNA is hypothesized to be regulators of macrophage M2 polarization [27]. Furthermore, Wu et al. evidenced that lncRNA-PVT1 was highly expressed in M2 macrophage-derived exosomes and regulated Th17 cell response in autoimmune encephalomyelitis [25]. Our results are consistent with these observations; the qRT-PCR results showed that PVT1 was significantly increased in M2 macrophages. Thus, lncRNA-PVT1 was confirmed as the preselected gene.

To the best of our knowledge, this was the first study to explore the role of Mg^2+^-doped CaSO_4_/β-TCP in regulating macrophage polarization and its relation with enhanced osteogenic differentiation and its regulating mechanisms. However, there are certain limitations in our study; one of the limitations is that the study focuses on the in vitro characterization of the mRNA and lncRNAs involved in macrophage polarization; therefore, further in vivo studies are needed the to gain more insight and prove the hypothesis.

## Conclusion

In conclusion, the present study elucidated that the osteoinductive capacities of Mg^2+^-doped CaSO_4_/β-TCP composite mediate through macrophage polarization and the osteogenic differentiation of periodontal ligament stem cells might be due to the presence of Mg^2+^. Furthermore, these findings will support the formulation of new strategies to design osteoinductive biomaterials by optimizing their physicochemical characteristics to endow them with favorable immunomodulatory properties in promoting periodontal bone regeneration.

## Supplementary Information

Below is the link to the electronic supplementary material.

Supplementary file1 (PDF 338 KB)

## Abbreviations

PD: Periodontal disease
CaSO_4_: Calcium sulfate and beta-tricalcium phosphate
β-TCP: Beta-tricalcium phosphate
SEM: Scanning electron microscopy
XRD: X-ray diffraction
TEM: Transmission electron microscope
ELISA: Enzyme-linked immunosorbent assay
qRT-PCR: Quantitative real-time polymerase chain reaction
GADPH: Glyceraldehyde-3-phosphate dehydrogenase
CD68: Cluster of differentiation 68
CD206: Cluster of differentiation 206
iNOS: Inducible nitric oxide synthase
