# Supplementary material for: Role of magnesium-doped calcium sulfate and β-tricalcium phosphate composite ceramics in macrophage polarization and osteo-induction
Source: Odontology. 2022 Jun 2;110(4):735–46. doi: 10.1007/s10266-022-00708-6 (PMC9463206; doi:10.1007/s10266-022-00708-6)
Supplement: Supplementary file 1 — Supplementary file1 (PDF 338 KB) [file 10266_2022_708_MOESM1_ESM.pdf]

Role of Magnesium Doped Calcium Sulphate and β- Tri-calcium Phosphate Composite Ceramics in Macrophage polarization and osteo-induction

Jing Zhou[1#], Su Sun[2#] Yan He[3], Tingting Yan[4], Jianfeng Sun[5], Jie Pan[6], Shuyu Zhu[7], Liqiong Chen[8], Pengfei Zhu[1], Xianghong Yang[1*], Yan Liu[8*]

[1] Department of Stomatology, Kunming Yanan hospital; KunMing,650051, China.
[2] Department of Stomatology, Kunming Municipal Hospital of Traditional Chinese Medicine, China.
[3] Laboratory for Regenerative Medicine, Tianyou Hospital, Wuhan University of Science and Technology, Wuhan, 430064, China.
[4] Faculty of Material Science and Engineering, Kunming University of Science and Technology,
Kunming, China.
[5] Department of Orthodontics, Ningbo Stomatological Hospital, Ningbo City, Zhejiang Province,
China.
[6] Department of Stomatology, The First People's Hospital of Yunnan Province, China.
[7] School of Stomatology, Kunming Medical University, China.
[8] The Second outpatient department, The Affiliated Stomatology Hospital of Kunming Medical University, China.

[#] **C**ontributed equally to the manuscript

***Corresponding Author:**

Xianghong Yang
Department of Stomatology, Kunming Yanan hospital,
No. 245 people's East Road, Kunming, China
E-mail: yangxh62@outlook.com

Yan Liu
The Second outpatient department,
The Affiliated Stomatology Hospital of Kunming Medical University
No. 37-3, Yuantong street, Wuhua District, Kunming, China.
Telephone: 0871-63211142; Fax 0871-63211143
E-mail: Lydyc@aliyun.com

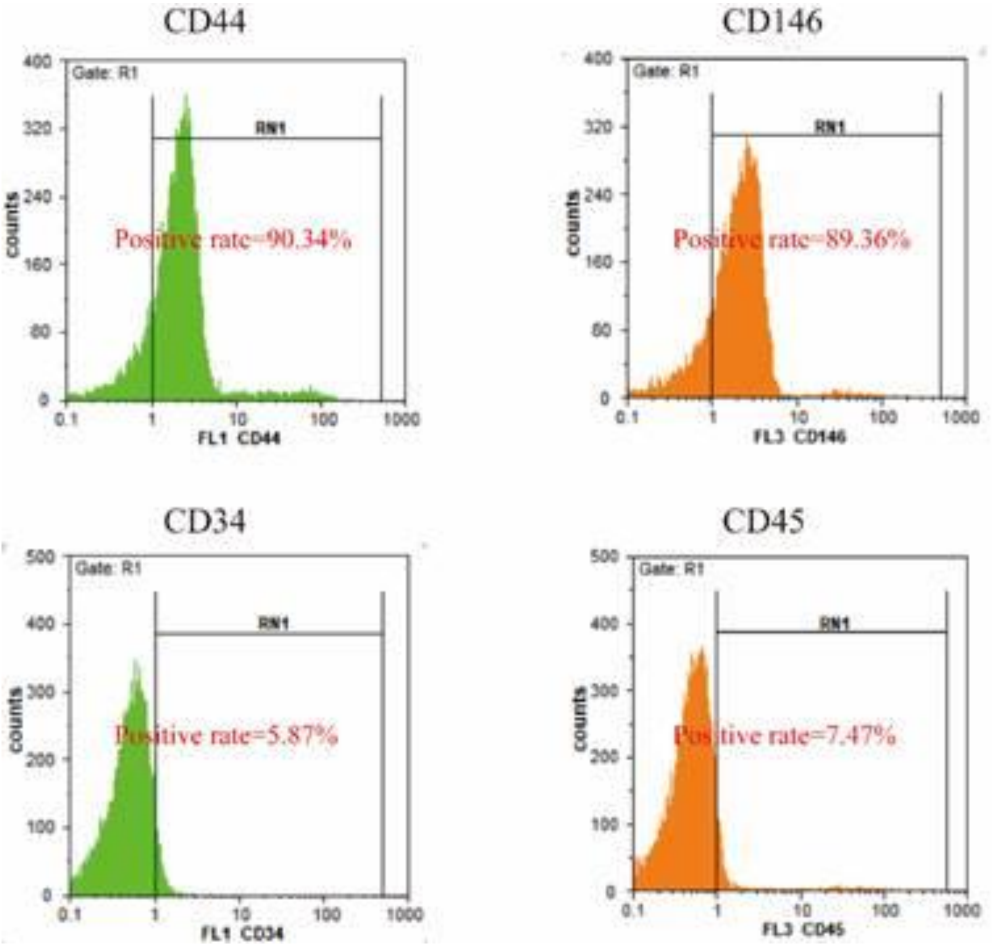

Online Resource 1 Evaluation of cytokine expression of periodontal ligament cells by flow cytometric analysis CD44: cluster of differentiation 44; CD45: cluster of differentiation 45; CD34: cluster of differentiation 34; CD146: cluster of differentiation 146

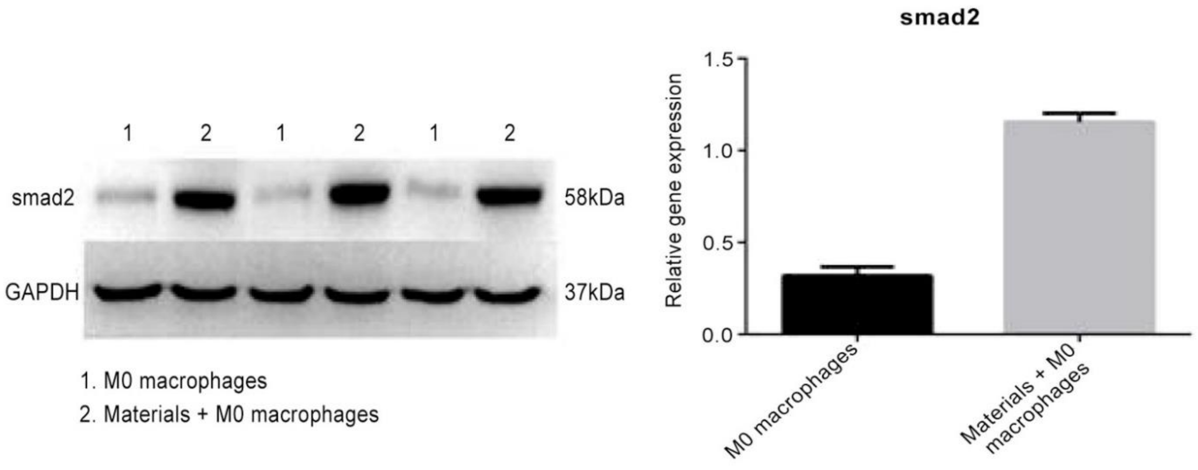

(a) (b)

Online Resource 2 a) Western Blot analysis of smad2 protein at 58 kDa b) Quantitative gene expression of the fold change of smad2 protein as compared to housekeeping GAPDH protein

GADPH: Glyceraldehyde-3-phosphate dehydrogenase

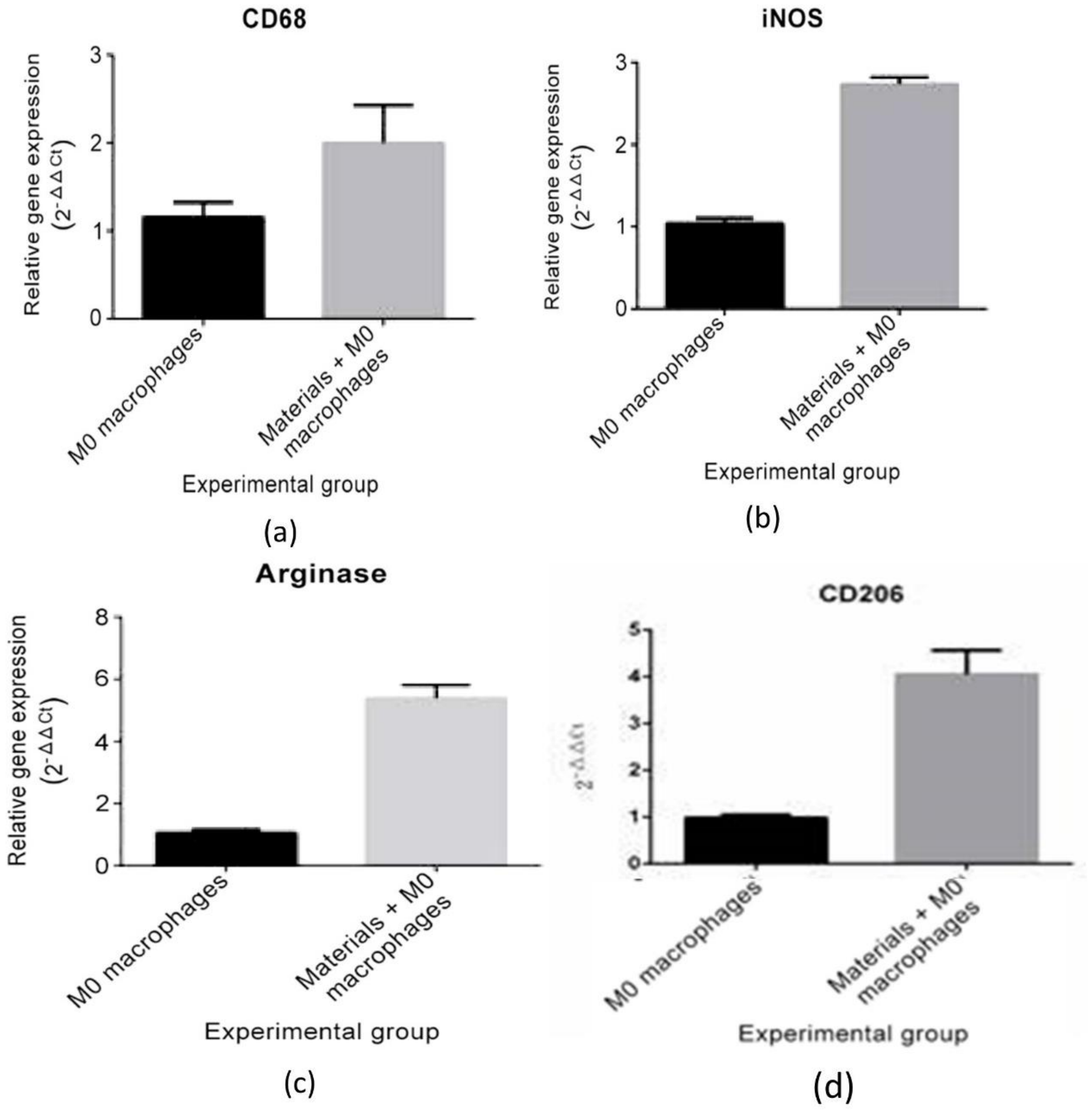

Online Resource 3 Relative gene expression of a) iNOS, b) CD68, c) arginase, d) CD206 markers by qRT-PCR CD68: cluster of differentiation 68; CD206: cluster of differentiation 206; inducible nitric oxide synthase (iNOS);

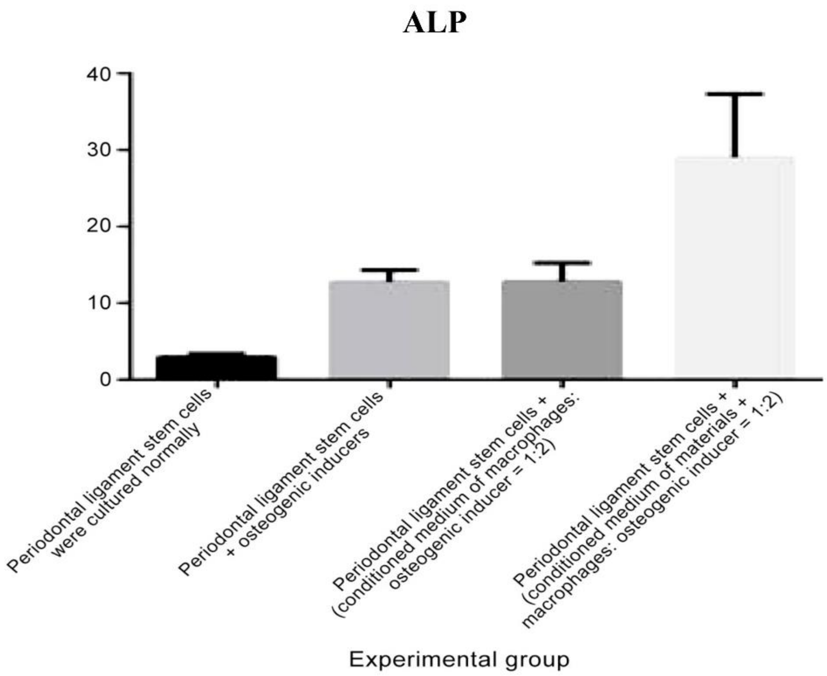

Online Resource 4 Detection of periodontal gene expression for co-cultured ($CaSO_4$)/β-TCP by ALP
